# Are Wild Prey Sufficient for the Top Predators in the Lowland Protected Areas of Nepal?

**DOI:** 10.1002/ece3.70387

**Published:** 2024-10-08

**Authors:** Saneer Lamichhane, Abhinaya Pathak, Aasish Gurung, Ajay Karki, Trishna Rayamajhi, Ambika Prasad Khatiwada, Jeffrey Mintz, Sudip Raj Niroula, Chiranjibi Prasad Pokharel

**Affiliations:** ^1^ Nepal Conservation and Research Center Ratnanagar, Chitwan Nepal; ^2^ Department of Wildlife Ecology and Conservation, School of Natural Resources and Environment University of Florida Gainesville Florida USA; ^3^ Department of National Parks and Wildlife Conservation, Babar Mahal Kathmandu Nepal; ^4^ Department of Ecology, Behavior and Evolution, School of Biological Sciences University of California San Diego California USA; ^5^ National Trust for Nature, Khumaltar Kathmandu Nepal; ^6^ Department of Zoology and Physiology, Haub School of Environment and Natural Resources University of Wyoming Laramie Wyoming USA; ^7^ Department of Natural Resources and the Environment Cornell University Ithaca New York USA; ^8^ Mahendra Morang Adarsha Multiple Campus Biratnagar Nepal

**Keywords:** canonical correlation, predator biomass, predator–prey interactions, predator–prey power law, prey biomass

## Abstract

A balanced equilibrium between carnivores and their prey is crucial for maintaining ecosystem sustainability. In this study, we applied the predator–prey power law equation to assess the balance between the biomass densities of carnivores and their wild prey within Nepal's lowland protected areas during 2013, 2018, and 2022. The estimated value of the power law exponent *k* for predator–prey biomass was 0.71 (95% CI = 0.39–1.05), indicating an approximate threefold increase in predator biomass density for every fivefold increase in prey biomass density. Consequently, this creates a systematically bottom‐heavy predator–prey biomass pyramid. This finding, consistent with the *k* = 3/4 trophic biomass scaling across ecosystems, suggests that predator biomass is proportionally sustained by prey biomass, indicating a balance between top predators and their wild prey in Nepal's lowland protected areas. We further demonstrated it is possible to retain the overall power law exponent while jointly measuring intraguild competition between two predators with canonical correlation analysis. This understanding opens avenues for future research directed toward unraveling the factors that drive these consistent growth patterns in ecological communities.

## Graphical abstract

The study in Nepal's lowland protected areas found a balanced predator–prey relationship, with a power law exponent “*k*” of 0.7 indicating a significant increase in predator biomass density with rising prey density. This sublinear growth pattern aligns with established trophic biomass scaling estimates, emphasizing its importance for stabilizing predator–prey dynamics and guiding future ecosystem management and research efforts.

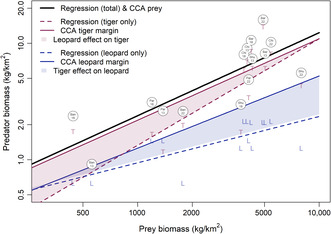

## 1 Introduction

The global extinction crisis has severely impacted terrestrial ecosystems, particularly affecting large mammals, many of which now face the imminent threat of extinction (Hilton‐Taylor 2000; Ceballos, Ehrlich and Raven 2020). Large carnivores are particularly vulnerable, with a significant portion at risk of extinction and experiencing population declines (Wolf and Ripple 2018). This vulnerability is exacerbated by habitat loss, illegal hunting, prey depletion, and human conflicts (Crooks 2002; Inskip and Zimmermann 2009). As apex predator, large carnivores are crucial for maintaining the functional integrity of diverse ecosystems (Ripple et al. 2014). For example, they influence and can regulate prey populations by imposing top‐down control and restricting mesopredators through interference competition, highlighting the importance of their conservation efforts (Hoeks et al. 2020).

Without an ample presence of prey species, populations of large carnivores can dwindle, potentially leading to local extinction. Additionally, the body size of available prey plays a significant role. While small prey species such as barking deer (*Muntiacus vaginalis*, 20 kg) may provide sufficient food for tigers (*Panthera tigris tigris*) when large prey like sambar (*Cervus unicolor*, 212 kg) are absent, raising a cub solely on small prey species alone is impractical (Kapfer et al. 2011; Kelchtermans 2020). A strong correlation exists between prey and carnivore abundance—approximately 10,000 kg of prey supporting approximately 90 kg of large carnivores within the Carnivora order (Carbone and Gittleman 2002)—making the abundance of prey species essential for the survival and existence of large carnivore populations (Kapfer et al. 2011; Pun et al. 2022; Carbone et al. 1999). Consequently, a crucial question for managers is whether prey populations within forest‐protected areas are adequate to sustain predator populations.

Besides sustaining carnivore populations, sufficient prey plays a crucial role in mitigating conflict between large carnivores and livestock (Berger, Buuveibaatar, and Mishra 2013; Wolf and Ripple 2016; Davoli et al. 2022). Research indicates that large carnivores avoid livestock when prey populations are adequate (Biswas and Sankar 2002; Reddy, Srinivasulu, and Rao 2004). Furthermore, when sufficient prey populations are present, the need to range far for food is reduced, thereby reducing negative interactions with humans such as mortality on roads (Hill, DeVault, and Belant 2021), direct persecution in regions with high human densities (Cardillo et al. 2004), and non‐consumptive human impacts on prey–predator spatial dynamics (Lamichhane et al. 2023). Conversely, when food resources are abundant beyond an animal's range, the chances of crop raiding or encroaching on human communities by prey species increase, as predators push these prey species closer to human settlements. This displacement leads to significant economic damages, typically outweighing losses due to livestock depredation by predators. For example, economic damages caused by wild boar in Europe reach €80 million/year (Valente et al. 2020; Reimoser and Putman 2011). Hence, it is imperative to understand the delicate balance between carnivores and their prey to foster coexistence between humans and wildlife.

A balanced equilibrium between carnivores and their prey is essential for ecosystem sustainability. Emphasis on charismatic carnivores, exemplified by the significant increase in Nepal's tiger population since 2010, may inadvertently lead to conflict with humans and livestock without sufficient prey (DNPWC and DFSC 2022; Habib et al. 2015). Hatton et al. (2015) revealed a general formula relating predator and prey trophic biomass, expressing it as a consistent scaling law applicable to terrestrial and aquatic biomes. Such a recurring three‐fourth relationship in the predator–prey biomass pyramid reflects the universal principles governing the interplay between predator and prey biomass across diverse ecosystems (Frank 2009; Marquet et al. 2005).

The objective of this study is to examine if the predator–prey power law equation holds in Nepal's lowland protected areas and assess the relationship between carnivore and wild prey biomass. A consistent relationship suggests ecosystem health, while deviations may indicate underlying issues such as disturbances or imbalances—a shallower slope, for instance, could signify local perturbations (e.g., over‐exploitation) where predator populations do not increase proportionally with prey (Oksanen and Oksanen 2000; Hatton et al. 2015; Perkins et al. 2022). Conversely, a steeper slope indicates top‐down controls of predators on prey, potentially leading to issues like livestock depredation (Berger, Buuveibaatar, and Mishra 2013; Hatton et al. 2015). These insights are valuable for conservation managers and researchers, underscoring the importance of maintaining the delicate equilibrium between predator and prey populations for sustainable ecosystem health.

We additionally applied the predator–prey power laws for leopards and tigers individually, and jointly through canonical correlation analysis (CCA, Hotelling 1936). CCA is a widely used multivariate statistical procedure capable of fitting correlated linear relationships between two sets of variables, in this case, between the two primary predator biomasses and the total prey biomass. It provides a bivariate relationship between the two predators, from which we are able to derive univariate marginal relationships for individual predator components. These marginal relationships may be of practical value for comparison against the current estimated predator species biomass densities to quantify competition between predators.

## 2 Materials and Methods

### 2.1 Study Area

**FIGURE 1:**
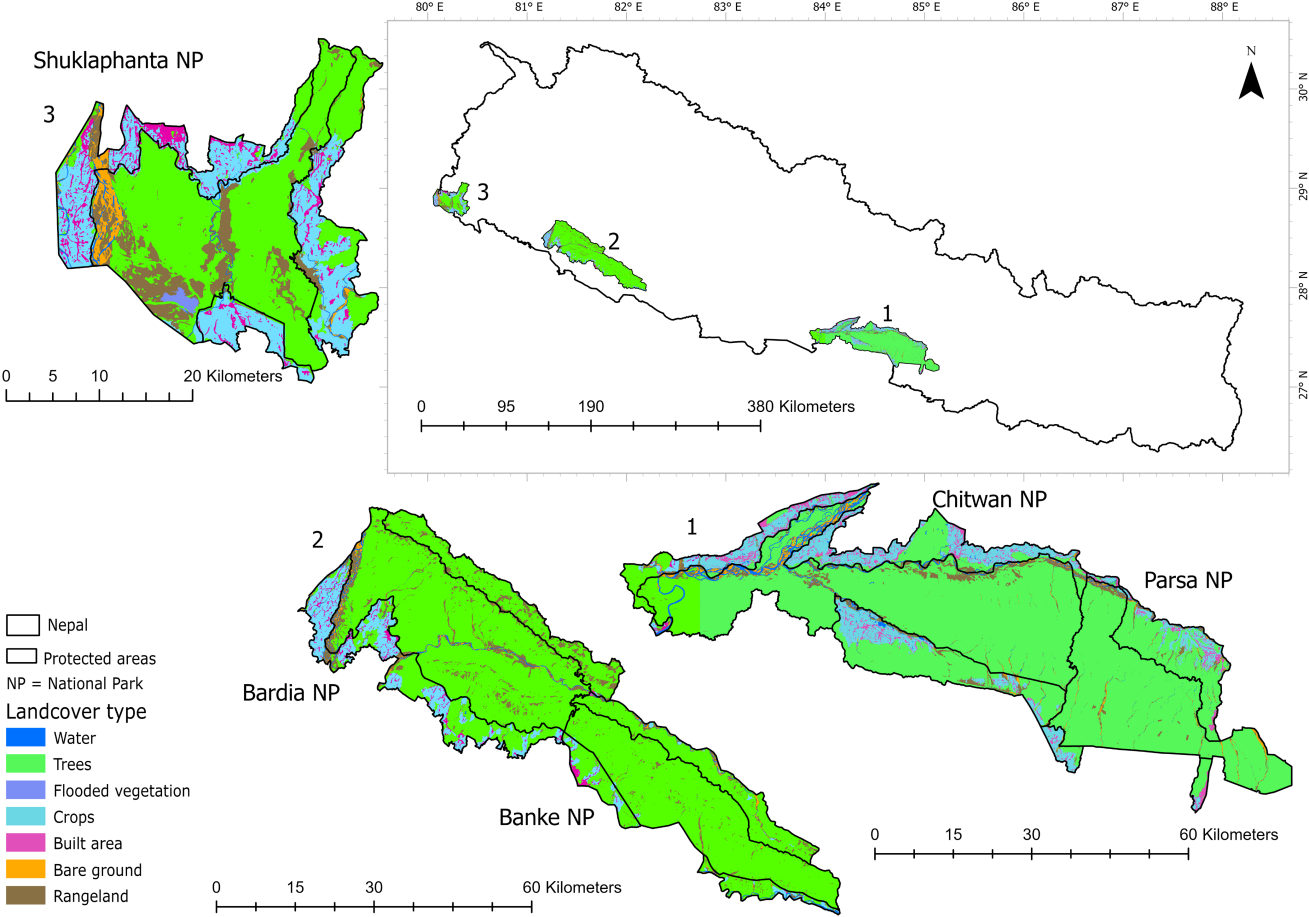
Study area map showing the five national parks in the lowlands of Nepal. The adjoining Parsa and Chitwan National Parks in the east, and Banke and Bardia National Parks in the mid‐west are numbered 1 and 2, respectively, and Shuklaphanta National Park in the far west is numbered 3. The land cover types of these national parks were prepared using the Esri|Sentinel‐2 10 m land use/land cover data for the year 2022 (Karra et al. 2021) using ArcGIS Pro (version 3.1.3) (Esri 2024).

Our study area encompasses five lowland protected areas (PAs) in Nepal renowned for their significant populations of large carnivores, specifically tigers and leopards (*Panthera pardus*). These PAs include Parsa National Park, Chitwan National Park, Banke National Park, Bardia National Park, and Shuklaphanta National Park (Figure 1). Legally, each of these PAs is separated into core and buffer zones. Human activities within the core area are restricted; entry to the parks requires permission from the national park office. The buffer zone encompasses the surrounding area including forests, agricultural lands, settlements, village open spaces, and various other land uses. The primary objective of the buffer zone is to achieve sustainable nature conservation while enabling local people to use forest resources to enhance their economic circumstances (HMGN (His Majesty's Government of Nepal) 1996; DNPWC 2023).

### 2.2 Data Collection

For each lowland protected area in Nepal, tiger densities (per 100 sq. km) and prey density (per sq. km) data were obtained from national tiger survey and line transect estimates conducted in 2013, 2018, and 2022 by the Department of National Parks and Wildlife Conservation (DNPWC), Nepal (Dhakal et al. 2014; DNPWC and DFSC 2018, 2022) (Table 1). Leopard densities (per 100 sq. km) for the closest corresponding years and regions were referenced from various literature sources (Thapa et al. 2014; Kandel, Lamichhane, and Subedi 2020; Wegge et al. 2009; Lamichhane et al. 2019; Pokheral and Wegge 2019). Dhole (*Cuon alpinus*), an endangered breed of local wild canid is believed to be present in all parks except Shuklaphanta National Park; however, dhole numbers are relatively low and their density is unknown in these national parks, so it was excluded from the primary analysis. All species densities were standardized per sq. km for uniform comparison across parks. The average weight of combined prey species in each national park was calculated by averaging the weights of individual prey species [sambar (*Rusa unicolor*, 212 kg), spotted deer (*Axis axis*, 55 kg), hog deer (*Axis porcinus*, 40 kg), wild boar (*Sus scrofa*, 38 kg), barking deer (*Muntiacus vaginalis*, 20 kg), langur (8 kg), and rhesus monkey (*Macaca mulatta*, 8 kg)] as reported in studies by Lamichhane and Jha (2015) and Pun et al. (2022). Similarly, the average weights of tigers (180 kg) and leopards (42 kg) were sourced from Pun et al. (2022) and Seidensticker (1976). To estimate the total biomass per sq. km for prey and predator species in each national park across 2013, 2018, and 2022, their respective densities (per sq. km) were multiplied by their average weights (Appendix S1).

**TABLE 1:** National Parks of the Nepali lowlands.

National park	Established year	Core, km^2^	Buffer, km^2^	Mammal species	Tiger density^f^, per 100 km^2^ (SE)	Prey density^f^, $\mathrm{per}\;km^{2}\;\left(\mathrm{SE}\right)$
Parsa (PNP)	1984	627.39	285.30	37^a^	1.74 (0.17)	75 (11.4)
Chitwan (CNP)	1973	952.63	729.37	68^b^	4.06 (0.22)	100 (9.1)
Banke (BaNP)	2010	550.0	343.0	34^c^	0.97 (0.12)	33 (6.6)
Bardia (BNP)	1976	968.0	327.0	60^d^	7.15 (0.38)	90 (11.2)
Shuklaphanta (ShNP)	1976	305.0	243.5	56^e^	1.99 (0.27)	146 (19.0)

*Note:* Tiger density and large prey density are reported for 2022, based on surveys by DNPWC and DFSC (2022). The line transect survey identified a diverse range of wild prey species in these areas, including four deer species (spotted deer, sambar, hog deer, and barking deer), two antelopes (blue bull and four‐horned antelope), wild boar, gaur, and two primate species (rhesus monkey and langur). Density information for 2013 and 2018 is provided in the data supplement.

^a^ PNP (2018).

^b^ CNP (2013).

^c^ BaNP (2018).

^d^ BNP (2022).

^e^ ShNP (2023).

^f^ DNPWC and DFSC (2022).

### 2.3 Analytical Approach

The predator–prey biomass power law describes how total biomass is distributed across communities at various trophic levels in the food chain (Trebilco et al. 2013; Cebrian et al. 2009; Chase et al. 2000). Power laws are simple functions of the form $y=c\cdot x^{k}$, where c is a coefficient (*y* value at *x* = 1) and *k* is the dimensionless scaling exponent (Frank 2009; Marquet et al. 2005). On logarithmic axes, power laws follow a straight line with a slope *k* (or a parameter beta of linear regression) but ordinary axes may curve up (*k* > 1) or down (*k* < 1).

The slope *k* of the relation of the log of predator biomass density (*y*) versus the log of prey biomass density (*x*) identifies the relative change in the shape of the pyramid. An exponent *k* > 1 means that the pyramid becomes relatively more top‐heavy at higher biomass and is predicted by top‐down control of predators on prey. A top‐heavy biomass pyramid can stabilize the ecosystem by regulating prey population through predation and mesocarnivores through intraguild competition, thereby maintaining biodiversity (Chase et al. 2000; Oksanen and Oksanen 2000; Estes et al. 2011; Hatton et al. 2015). An exponent *k* = 1 indicates that the pyramid shape remains constant and is predicted by bottom‐up control, whereby a constant fraction of biomass is produced and transferred to each successively higher trophic level (Leibold et al. 1997; Arditi and Ginzburg 2012; Hatton et al. 2015). Values of *k* < 1 indicate that the pyramid becomes relatively more bottom‐heavy at higher biomass, indicating a lack of sufficient predator populations to control prey numbers, favoring overgrazing and habitat degradation (Ripple et al. 2014). Hatton et al. (2015) revealed that across ecosystems, highly consistent pyramid size, shape, and growth patterns exist, and community trophic biomass scaling is commonly near *k* = ¾.

We employed a Bayesian approach using JAGS (Just another Gibbs sampler—Plummer 2015) in R to investigate the predator–prey power law relationship for the combined biomass of predators and prey. The fundamental power law equation $y=c\cdot x^{k}$ was log‐transformed to $\log\left(y\right)=\log\left(c\right)+k\cdot \log\left(x\right)$, allowing us to express the model within a linear regression framework. First, we simulated a test dataset of the log relation between predator biomass per square km (*y*) as a function of predictor variable prey biomass per square km (*x*). We fit a standard Bayesian linear regression for the log‐transformed biomass densities, with priors for coefficients $\log\left(c\right)$ and *k* normally distributed. The model was able to successfully recover the simulated parameters for $\log\left(c\right)$ and *k*, so we proceeded to apply the model to the observed biomass densities. Three linear models of this form were created for comparison: a total predator biomass density regression, a tiger‐only biomass density regression model, and a leopard‐only biomass density regression model.

The relationship between tiger and leopard biomass and their shared prey biomass was examined jointly using a traditional multivariate technique, canonical correlation analysis (CCA, Hotelling 1936). CCA seeks the most correlated linear relationships across two datasets. Due to the logarithmic nature of the predator–prey power law, we first split the total prey multiplicatively into an effect of tiger biomass alone, T, and the quantity $\left(T+L\right)/T$, which we interpret as the relative increase in total biomass when leopards are also included. For instance, if the biomass of tiger and leopard were equal, the total biomass would be twice as large when leopards are included. When log is applied, the leopard component may also be interpreted as the multiplicative effect of leopard predation after accounting for tiger predation, $\log\left(T+L\right)-\log\left(T\right).$

$$\log\left(T+L\right)=\log\left(T\cdot \frac{T+L}{T}\right)=\log\left(T\right)+\log\left(\frac{T+L}{T}\right)$$

Canonical correlation analysis allows us to obtain linear relationships among the biomass of tigers and leopards which are maximally correlated with the log biomass of prey.

$$\rho =\mathrm{cor}\left(\alpha_{1}\log\left(T\right)+\alpha_{2}\log\left(\frac{T+L}{T}\right),\;k\cdot \log\left(x\right)\right)$$

**FIGURE 2:**
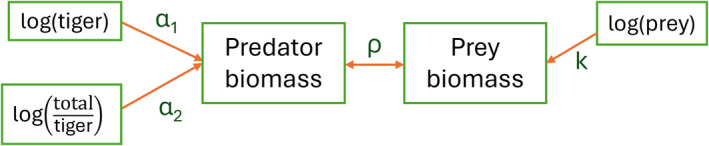
Diagram representing the relationships between variables in canonical correlation analysis. The fitted predator biomass linear relationship had a correlation of *ρ* = 0.81 with the total prey biomass linear relationship.

In general, the correlation ρ of the regression of y on x is related to the slope β by $\rho =\beta \cdot \frac{\sigma_{x}}{\sigma_{y}}$ (Burlington and May 1953). In the case of CCA, the standard deviations of the linear combinations match $\left(\sigma_{x}=\sigma_{y}\right)$, therefore the two linear relationships will be related through the correlation between them (Figure 2):

$$\alpha_{1}\log\left(T\right)+\alpha_{2}\log\left(\frac{T+L}{T}\right)=\rho \cdot k\cdot \log\left(x\right)$$

Our primary interest lies is in interpreting the implications on predator–prey relationships for tigers and leopards with respect to prey biomass. To do this, we consider the prey biomass density exponent when tiger or leopard make up nearly the entire population, which forms the margin of the bivariate relationship between leopard and tiger. For example we can examine the tiger's marginal relationship when there are no leopards (L=0), or the leopard's margin when the minimum number of tigers are present (T=1).

## 3 Results

**FIGURE 3:**
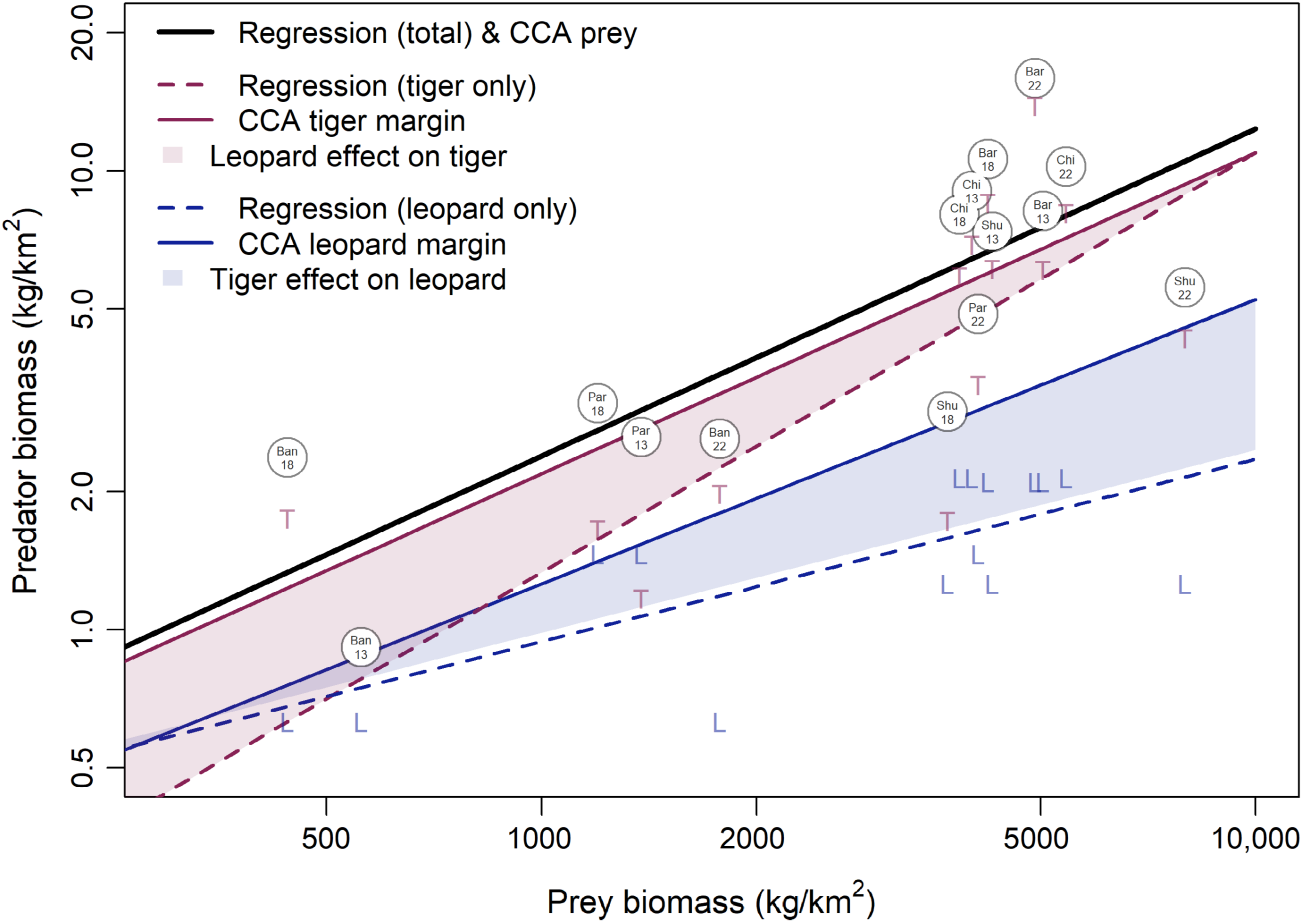
Predator–prey power models of total predator (circles), individual tiger (T), or leopard (L) biomass densities. The canonical correlation analysis (CCA) linear relationship for prey biomass density lies coincident with the regression model for the total prey (black solid line). Predator margins are the CCA‐estimated biomass density attained for leopard and tiger in the absence of the other competitor (solid blue and red lines). Tiger‐ and leopard‐only regression models are the observed behavior of each species in the presence of the second species (dashed lines). The difference between the margin and the individual species regression (shaded) is interpreted as competition. Further illustrations showing differences by location and year are available in Appendix B.

**FIGURE A1:**
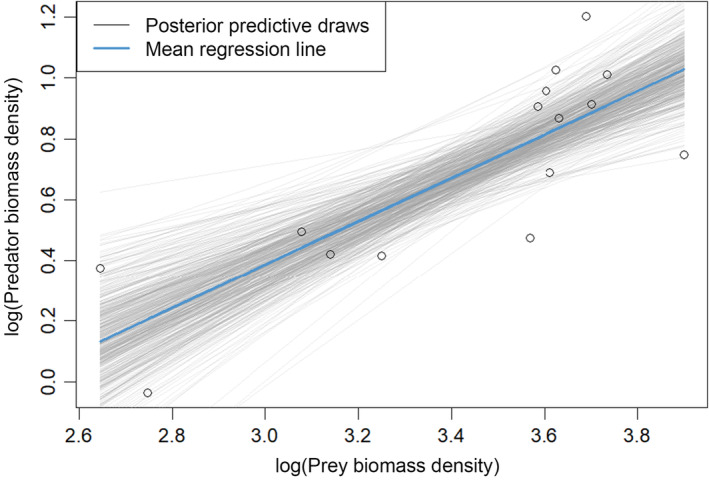
A posterior predictive linear regression plot depicting the model's predictions based on Markov chain Monte Carlo (MCMC) samples. The black dots represent the observed data points, and the blue line represents the mean regression line computed from the MCMC samples. The light gray lines show individual predictions generated from different MCMC samples, providing a sense of prediction uncertainty. The model was trained on log‐transformed prey biomass density (*x*) to predict log‐transformed predator biomass density (*y*).

**FIGURE A2:**
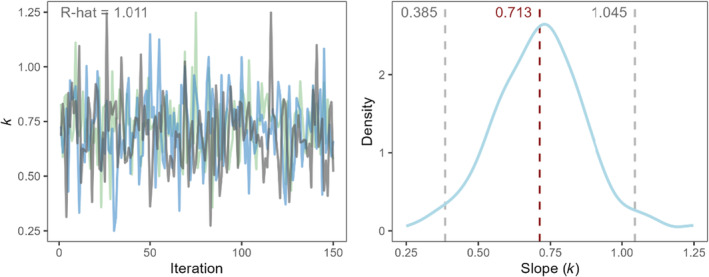
(Left) A traceplot illustrating the Markov chain Monte Carlo (MCMC) sampling of *k* (or parameter beta) over 10,000 iterations, with thinning of 50×. Each colored line represents a separate chain. The *y*‐axis displays sampled values of beta, and the *x*‐axis represents the iteration number. Convergence and mixing of chains appear satisfactory, as evidenced by the absence of systematic patterns. A burn‐in period of 2,500 iterations was discarded for stabilization. (Right) A density plot for the posterior distribution of parameter “*k*” obtained from Markov chain Monte Carlo (MCMC) simulations. The plot showcases the distribution's shape, providing insights into parameter uncertainty. The light blue curve is a Gaussian kernel density estimate for “*k*”, offering a smoothed representation of the underlying distribution. The vertical red line denotes the mean value of “*k*”, and the gray dashed lines indicate the 95% credible interval, offering a range of plausible parameter values.

**FIGURE A3:**
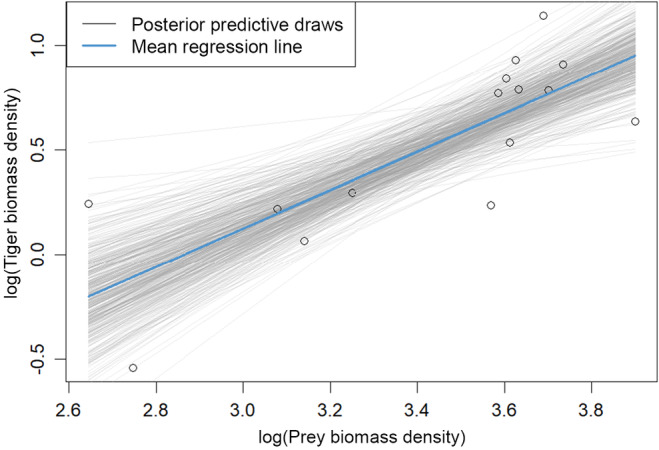
A posterior predictive linear regression plot for tiger‐only model.

**FIGURE A4:**
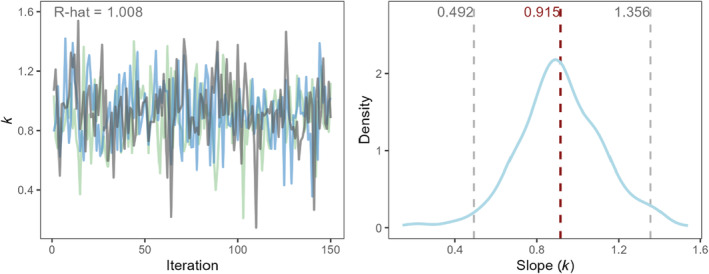
(Left) Traceplot from fitting the tiger‐only model, illustrating the MCMC sampling of *k* for the remainder of 10,000 iterations, after a burn‐in period of 2,500 iterations and thinning of 50×. Each colored line represents a separate chain. (Right) Density plot for the posterior distribution of parameter “*k*” obtained for the tiger‐only regression model.

**FIGURE A5:**
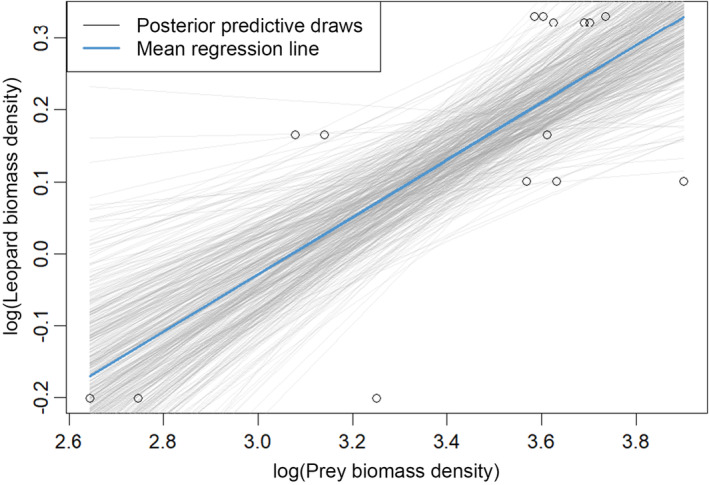
A posterior predictive linear regression plot for leopard‐only model.

**FIGURE A6:**
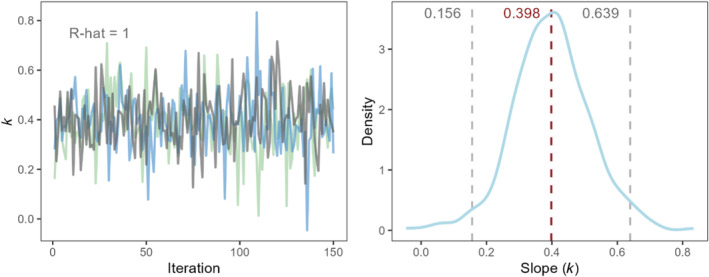
(Left) Traceplot from fitting the leopard‐only model, illustrating the MCMC sampling of *k* for the remainder of 10,000 iterations, after a burn‐in period of 2,500 iterations. Each colored line represents a separate chain. (Right) Density plot for the posterior distribution of parameter “*k*” obtained for the leopard‐only regression model.

The parameter *k* in the predator–prey power law was estimated to be 0.71 (95% CI = 0.39–1.05). For the tiger‐ and leopard‐only regressions, *k* was 0.95 (0.95% CI = 0.49–1.36) and 0.40 (95% CI = 0.16–0.64) (Appendix A: Figures A1, A2, A3, A4, A5, A6). The power coefficient estimated by canonical correlation analysis, *k* = 0.71, exactly matched that obtained from regression for the overall power law; however, no confidence interval is immediately available for the coefficients of the linear combinations in CCA. According to Mardia, Kent, and Bibby (1979), testing for the significance of the individual coefficients of the linear relationships fit by CCA has proven to be difficult. For the predator side linear combination, the coefficients were $a_{1}=0.83$ and $a_{2}=0.93$. The predator and prey linear relationships had a correlation of ρ=0.81, the significance of which can be tested by applying a Wilks' Λ test, $p\left(\chi_{2}\geq 12.8\right)=0.0017$ (Figure 3).

**FIGURE A7:**
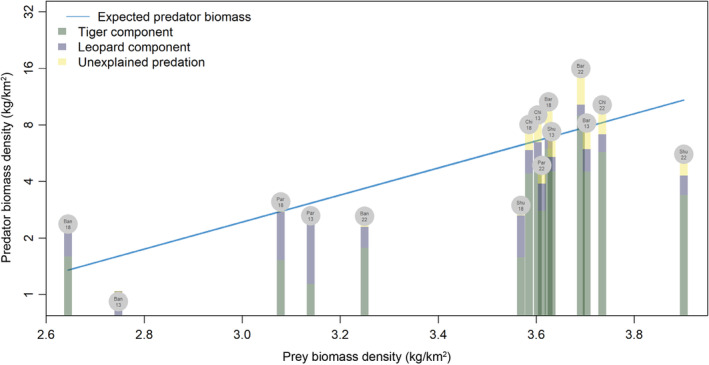
Contributions of leopard and tiger to total predation. Estimates of tiger and leopard contributions to predation based on the first canonical variates, with contributions of tigers shown in vertical green, and the contribution of leopards in blue. Leopard contribution is parameterized as the relative increase (multiplicatively) in total predator biomass when leopards are included. Yellow represents components of total predation not accounted for by the linear combination obtained from CCA.

**FIGURE A8:**
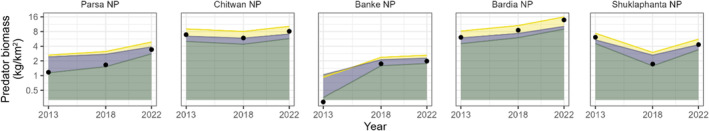
Estimates of predation components over time by tiger (green), leopard (blue), and predation unaccounted for by canonical correlation analysis. Nepal's national parks (NP) are listed in order from east (on left) to west.

The rate of gain in log tiger biomass density when leopard was held to zero, or tiger margin, was estimated to be k=0.93. For leopards, the slope of the margin was k=0.83, indicating the rate of gain in the leopard component when the tiger biomass density was held to a relatively low value of $1\;\mathrm{kg}/km^{2}$ (the lowest observed tiger density reported was $0.288\;\mathrm{kg}/km^{2}$ in Banke NP during 2013). For more information on the calculation of the margins and their sensitivity, refer to Appendix B (Figures A7 and A8) or code supplement Appendix S2.

## 4 Discussion

Our findings reveal an approximately threefold increase in predator biomass for every fivefold rise in prey biomass (*k* = 0.71, 95% CI = 0.4–1). Consequently, as the total biomass increases, the shape of the predator–prey biomass pyramid systematically shifts toward a bottom‐heavy configuration (*k* < 1). These observed patterns at the ecosystem level quantitatively corroborate prior analyses of predator–prey biomass scaling across trophic groups which identified sub‐linear scaling with *k* values ranging between 0.66 and about 0.76 (Perkins et al. 2022; Hatton et al. 2015; McCauley and Kalff 1981), but are higher than the overall value of 0.33 reported by Chatterjee et al. (2023). Our results suggest that the predator–prey power law applies to the lowland protected areas of Nepal, and thus at equilibrium, predator biomass is proportionally sustained to prey biomass highlighting the balance between carnivore and their wild prey in Nepal's lowland protected areas (Hatton et al. 2015; Cebrian and Lartigue 2004).

To examine how each predator contributes to total predation, we fit individual predator–prey power relationships for tiger and leopard biomass against total prey biomass, analogous to constructing a model for one species, while being unaware of the importance of a secondary predator. These individual species regressions resulted in more extreme scaling coefficients (k=0.92 for tiger and k=0.39 for leopard). The change in the fitted predator–prey power coefficients reflects the dependence of modeled power coefficient *k* on the omitted predator species; for instance, omitting tigers, which attain high biomass density at sites where prey are abundant, resulted in a model that downplays the importance of prey biomass (lower slope). On the other hand, omitting leopards from a tiger biomass model resulted in lower baseline estimates of tiger biomass (intercept) and placed higher importance on increases in prey biomass for the growth of tiger biomass (slope). This trade‐off suggests the impact of omitting dhole will depend on whether dhole favor high or low prey biomass density sites. If dhole were evenly distributed, the effect would be similar to omitting leopard from a tiger model, increasing the estimate of *k* by failing to account for prey use by dhole within low biomass density sites.

Rather than omitting a species, canonical correlation analysis allowed us to jointly estimate predator relationships with total prey, while preserving the overall regression fit for prey (*k* = 0.71). The bivariate relationship fit for tiger and leopard had a tiger margin of *k* = 0.70 and leopard margin of *k* = 0.62. This results into higher biomass density estimates for tigers in the absence of leopards in low biomass sites (Figure 3). Leopard biomass density in the absence of tigers was estimated to rise more rapidly with prey biomass density, allowing leopards to achieve higher biomass densities at high prey‐density sites in the absence of tigers. For instance, leopard might achieve mean densities of 3–3.5 kg/km^2^ in Chitwan where prey density regularly ranges from 4 to 5,000 kg/km^2^, up around 50% from current observed leopard biomasses of 2.1 kg/km^2^, which provides some quantitative indication of the impact of tiger competition for prey on the densities of leopards in Chitwan.

In the lowland protected areas of Nepal, interspecific interaction is widespread between dominant tigers and subordinate leopards for principal prey species and space (Pun et al. 2022; Kandel, Lamichhane, and Subedi 2020; Lovari et al. 2015; Lamichhane and Jha 2015; Bhattarai and Kindlmann 2012; Odden, Wegge, and Fredriksen 2010). A substantial increase in the tiger population has occurred, from 121 (CI) to 355 (CI) over the last decade (2010–2022) (DNPWC and DFSC 2022), intensifying competition and negatively affecting leopards. These cats experience interference competition, where tigers directly alter the resource‐attaining behavior of leopards, and exploitative competition, where tigers use resources (space and prey) reducing availability for leopards (Begon, Harper, and Townsend 1996; Mondal et al. 2012). Consequently, more tolerant of human presence, leopards are driven into more disturbed areas, such as buffer zones, increasing the likelihood of human–leopard conflict (Odden, Wegge, and Fredriksen 2010). If protection measures allow prey populations to grow, one might expect a reduction in direct conflict between the predators. However, if prey becomes abundant but habitat space remains limited, competition for specific habitat types could intensify, with tigers occupying prime habitats and forcing leopards into suboptimal areas. Prey might adapt by using areas less favored by their primary predators to reduce predation risk, further complicating predator–prey dynamics and spatial distribution (Lamichhane et al. 2023). Over time, the stronger tiger competitor may eliminate the weaker leopard competitor through competitive exclusion (Hardin 1960). Thus, assessing leopard density within these parks is crucial for understanding trophic dynamics. While our research indicates proportional maintenance of predator–prey biomass, it is important to comprehend the interplay between intraguild competition, habitat‐specific competition, and human activities for effective predator management and conservation strategies. For instance, in 2021, tiger and leopard predation in Nepal's lowland PAs resulted in 24 human and 4,427 livestock casualties, with tigers alone causing 21 human and 481 livestock deaths, often linked to human activities like entering buffer zones for fodder collection and livestock grazing, highlighting the need for community awareness about predator behavior and ecology to mitigate future incidents (DNPWC 2022; Lamichhane et al. 2018).

Our study represents an initial exploration into predator–prey biomass scaling within intricate food webs in Nepal but has some notable limitations. Firstly, comprehensive data on the biomass of all basal resources were unavailable (e.g., dholes), so our analysis was focused on higher trophic predators (tigers and leopards) preying mostly on sizes > 20 kg. Secondly, we were restricted to analyzing three‐season data for each protected area. This limitation impedes our capacity to investigate longitudinal trends such as autocorrelation in biomass over time. Although we treated each park and year as independent, due to the temporal and spatial separation between protected areas, more sophisticated modeling techniques could enhance our ability to account for potential correlations. As additional survey data become available over the coming decade, further research will be needed to unravel the complexities of predator–prey relationships in diverse ecosystems, providing a foundation for informed conservation strategies and sustainable management practices.

To summarize, a consistent pattern is emerging in trophic structure in terrestrial ecosystems. The power law scaling in predator–prey biomass, alongside the regularity of these patterns, highlights a conserved link between how ecosystems are structured and how they function. The sublinear growth scaling identified contributes to stabilizing predator–prey interactions in the lowlands protected areas of Nepal, emphasizing the importance of understanding these dynamics for effective ecosystem management. This insight paves the way for future research endeavors aimed at modeling the specific factors including competition driving these consistent growth patterns in ecological communities.

## 5 Conclusion

Maintaining a balanced equilibrium between carnivores and their prey is fundamental for the enduring health and sustainability of ecosystems. A global pattern emerges, revealing consistent scaling laws across diverse terrestrial and aquatic biomes, particularly the recurrent three‐fourth relationship in the predator–prey biomass pyramid, illuminates a universal principle governing the dynamics of these interactions. Our study evaluated the predator–prey balance in Nepal's lowland protected areas using the predator–prey power law equation, which aligned with community estimates of trophic biomass scaling. The findings suggested that predator biomass was proportionally sustained by prey biomass, indicating a balanced relationship between top predators and their wild prey in these protected areas. We also observe that new insights such as competition among predators may be attained by applying multivariate statistics (CCA) with the predator–prey power law. Our findings carry significant implications for conservation practitioners and researchers alike, highlighting the imperative to comprehend and actively uphold the delicate balance between prey and predator populations, and emphasizing the interconnectedness of species dynamics within ecological communities. This understanding is crucial for effective conservation strategies that ensure the overall health and sustainability of ecosystems.

## Author Contributions

**Saneer Lamichhane:** conceptualization (lead), data curation (lead), formal analysis (lead), methodology (lead), validation (lead), writing – original draft (lead), writing – review and editing (lead). **Abhinaya Pathak:** data curation (equal), formal analysis (equal), validation (equal), visualization (equal), writing – original draft (equal). **Aasish Gurung:** conceptualization (equal), data curation (equal), methodology (equal), visualization (equal). **Ajay Karki:** conceptualization (equal), data curation (equal), supervision (equal), validation (equal), writing – original draft (equal). **Trishna Rayamajhi:** conceptualization (equal), investigation (equal), methodology (equal), software (equal), writing – original draft (equal). **Ambika Prasad Khatiwada:** conceptualization (equal), investigation (equal), validation (equal), writing – original draft (equal). **Jeffrey Mintz:** data curation (equal), formal analysis (equal), investigation (equal), methodology (equal), software (equal), validation (equal). **Sudip Raj Niroula:** conceptualization (equal), formal analysis (equal), writing – original draft (equal). **Chiranjibi Prasad Pokharel:** data curation (equal), formal analysis (equal), supervision (lead), validation (equal), writing – original draft (equal), writing – review and editing (equal).

## Conflicts of Interest

The authors declare no conflicts of interest.

## Permission to Reproduce Materials from Other Sources

The authors have nothing to report.

## Supporting information


Appendix S1

Appendix S2

## Data Availability

The data and code can be accessed via the Dryad repository: https://datadryad.org/stash/share/fPikEIoMOmtp5K5eFxgZ2gYIHczntnM9‐WJ9T2eTmvY.

## Appendix B

### B.1 Canonical Correlation Analysis, Predation Components

Within each site, we applied the α coefficients to partition the total predation into estimated contributions by tiger (vertical green) and leopard (vertical blue) within Figure A7. The linear combinations fit by CCA do not explain all of the variation in observed predation (points), therefore gaps remain between the points and both vertical bars representing the CCA predation components, and the light blue regression line representing the linear predator–prey relationship shared by CCA and regression.

Leave‐one‐out cross validation was applied to assess sensitivity and variation in fitted parameters within canonical correlation analysis, and we report the 95% quantiles for the statistics obtained. CCA resulted in linear combinations of predators and prey with a correlation of 0.81 (CI: 0.76–0.87). The slope of linear relationship for log prey was k=0.713 (95% CI: 0.717–0.861), in agreement with the *k* found through regression of the log of total predator biomass against log of prey biomass. Cross‐validation revealed the Banke NP 2013 and 2018 observations, which were the lowest biomass site‐years for both prey and predators, played an important role in estimating the remaining parameters. Therefore, we retained these points in all models but report the statistics obtained when omitting these sites individually as $b_{13}$ and $b_{18}$. The predator linear combination coefficients were $\alpha_{1}=0.83$ (95% CI 0.84–0.95; $b_{13}=1.06$, $b_{18}=0.31$) for the tiger component and $\alpha_{2}=0.93$ (95% CI 0.93–1.31; $b_{13}=1.31$, $b_{18}=-1.15$) for the leopard component. Through algebra, the marginal *k* for leopard when tiger was minimal was determined to be 0.81·0.713/0.93 = 0.62 (95% CI 0.46–0.63; $b_{13}=0.56$, $b_{18}=2.47$) and the marginal *k* for leopard when tiger was minimal obtained was 0.81·0.713/0.83 = 0.70 (95% CI 0.62–0.71; $b_{13}=0.46$, $b_{18}=-0.67$).

## References

[ece370387-bib-0001] Arditi, R. , and L. R. Ginzburg . 2012. How Species Interact: Altering the Standard View on Trophic Ecology. Oxford, UK: Oxford University Press.

[ece370387-bib-0002] BaNP . 2018. Banke National Park and Buffer Zone Management Plan (FY2076/76‐2079‐2080). Kathmandu, Nepal: Department of National Parks and Wildlife Conservation. Ministry of Forests and Environment.

[ece370387-bib-0003] Begon, M. , J. L. Harper , and C. R. Townsend . 1996. Ecology: Individuals, Populations and Communities. 3rd ed. New Jersey: Wiley‐Blackwell.

[ece370387-bib-0004] Berger, J. , B. Buuveibaatar , and C. Mishra . 2013. “Globalization of the Cashmere Market and the Decline of Large Mammals in Central Asia.” Conservation Biology 27, no. 4: 679–689. 23866036 10.1111/cobi.12100

[ece370387-bib-0005] Bhattarai, B. P. , and P. Kindlmann . 2012. “Interactions Between Bengal Tiger (*Panthera tigris*) and Leopard (*Panthera pardus*): Implications for Their Conservation.” Biodiversity and Conservation 21: 2075–2094.

[ece370387-bib-0006] Biswas, S. , and K. Sankar . 2002. “Prey Abundance and Food Habit of Tigers (*Panthera tigris tigris*) in Pench National Park, Madhya Pradesh, India.” Journal of Zoology 256, no. 3: 411–420.

[ece370387-bib-0007] BNP . 2022. Management Plan of Bardia National Park and Its Buffer Zone (FY2079/80‐2083‐2084). Kathmandu, Nepal: Department of National Parks and Wildlife Conservation. Ministry of Forests and Environment.

[ece370387-bib-0008] Burlington, R. S. , and D. C. May . 1953. Handbook of Probability and Statistics With Tables. Sandusky, Ohio, USA: Handbook Publishers, Inc.

[ece370387-bib-0009] Carbone, C. , and J. L. Gittleman . 2002. “A Common Rule for the Scaling of Carnivore Density.” Science 295, no. 5563: 2273–2276. 11910114 10.1126/science.1067994

[ece370387-bib-0010] Carbone, C. , G. M. Mace , S. C. Roberts , and D. W. Macdonald . 1999. “Energetic Constraints on the Diet of Terrestrial Carnivores.” Nature 402, no. 6759: 286–288. 10580498 10.1038/46266

[ece370387-bib-0011] Cardillo, M. , A. Purvis , W. Sechrest , J. L. Gittleman , J. Bielby , and G. M. Mace . 2004. “Human Population Density and Extinction Risk in the World's Carnivores.” PLoS Biology 2, no. 7: e197. 15252445 10.1371/journal.pbio.0020197 PMC449851

[ece370387-bib-0201] Ceballos, G. , P. R. Ehrlich , and P. H. Raven . 2020. “Vertebrates on the Brink as Indicators of Biological Annihilation and the Sixth Mass Extinction.” Proceedings of the National Academy of Sciences of the United States of America 117, no. 24: 13596–13602. 32482862 10.1073/pnas.1922686117 PMC7306750

[ece370387-bib-0012] Cebrian, J. , and J. Lartigue . 2004. “Patterns of Herbivory and Decomposition in Aquatic and Terrestrial Ecosystems.” Ecological Monographs 74, no. 2: 237–259.

[ece370387-bib-0013] Cebrian, J. , J. B. Shurin , E. T. Borer , et al. 2009. “Producer Nutritional Quality Controls Ecosystem Trophic Structure.” PLoS One 4, no. 3: e4929. 19300514 10.1371/journal.pone.0004929 PMC2654170

[ece370387-bib-0014] Chase, J. M. , M. A. Leibold , A. L. Downing , and J. B. Shurin . 2000. “The Effects of Productivity, Herbivory, and Plant Species Turnover in Grassland Food Webs.” Ecology 81, no. 9: 2485–2497.

[ece370387-bib-0015] Chatterjee, N. , I. Mukhopadhyay , P. Nigam , and B. Habib . 2023. “Predicting Carrying Capacity of a Large Carnivore From Prey Densities: A New Approach.” PeerJ 11: e15914. 38025689 10.7717/peerj.15914 PMC10676078

[ece370387-bib-0016] CNP . 2013. Chitwan National Park and Buffer Zone Management Plan 2013–2017. Kathmandu, Nepal: Department of National Parks and Wildlife Conservation. Ministry of Forests and Environment.

[ece370387-bib-0017] Crooks, J. A. 2002. “Characterizing Ecosystem‐Level Consequences of Biological Invasions: The Role of Ecosystem Engineers.” Oikos 97, no. 2: 153–166.

[ece370387-bib-0018] Davoli, M. , A. Ghoddousi , F. M. Sabatini , E. Fabbri , R. Caniglia , and T. Kuemmerle . 2022. “Changing Patterns of Conflict Between Humans, Carnivores and Crop‐Raiding Prey as Large Carnivores Recolonize Human‐Dominated Landscapes.” Biological Conservation 269: 109553.

[ece370387-bib-0019] Dhakal, M. , M. Karki (Thapa) , S. R. Jnawali , et al. 2014. Status of Tigers and Prey in Nepal. Kathmandu, Nepal: Department of National Parks and Wildlife Conservation.

[ece370387-bib-0020] DNPWC . 2022. Annual Report (2021–2022). Kathmandu, Nepal: Department of National Parks and Wildlife Conservation. Ministry of Forests and Environment.

[ece370387-bib-0021] DNPWC . 2023. “Department of National Parks and Wildlife Conservation.” Visited on 13th December 2023. https://dnpwc.gov.np/en/.

[ece370387-bib-0022] DNPWC and DFSC . 2018. Status of Tigers and Prey in Nepal. Kathmandu, Nepal: Department of National Parks and Wildlife Conservation & Department of Forests and Soil Conservation. Ministry of Forests and Environment.

[ece370387-bib-0023] DNPWC and DFSC . 2022. Status of Tigers and Prey in Nepal. Kathmandu, Nepal: Department of National Parks and Wildlife Conservation & Department of Forests and Soil Conservation. Ministry of Forests and Environment.

[ece370387-bib-0024] Esri . 2024. “*ArcGIS Pro* (Version 3.1) [Software].” https://www.esri.com/en‐us/arcgis/products/arcgis‐pro.

[ece370387-bib-0203] Estes, J. A. , J. Terborgh , J. S. Brashares , et al. 2011. “Trophic Downgrading of Planet Earth.” Science 333, no. 6040: 301–306. 21764740 10.1126/science.1205106

[ece370387-bib-0025] Frank, S. A. 2009. “The Common Patterns of Nature.” Journal of Evolutionary Biology 22, no. 8: 1563–1585. 19538344 10.1111/j.1420-9101.2009.01775.x PMC2824446

[ece370387-bib-0026] Habib, A. , I. Nazir , M. F. Fazili , and B. A. Bhat . 2015. “Human‐Wildlife Conflict‐Causes, Consequences and Mitigation Measures With Special Reference to Kashmir.” Journal of Zoology Studies 2, no. 1: 26–30.

[ece370387-bib-0204] Hardin, G. 1960. “The Competitive Exclusion Principle: An Idea that Took a Century to be Born has Implications in Ecology, Economics, and Genetics.” Science 131, no. 3409: 1292–1297. 14399717 10.1126/science.131.3409.1292

[ece370387-bib-0027] Hatton, I. A. , K. S. McCann , J. M. Fryxell , et al. 2015. “The Predator–Prey Power Law: Biomass Scaling Across Terrestrial and Aquatic Biomes.” Science 349, no. 6252: aac6284. 26339034 10.1126/science.aac6284

[ece370387-bib-0028] Hill, J. E. , T. L. DeVault , and J. L. Belant . 2021. “A Review of Ecological Factors Promoting Road Use by Mammals.” Mammal Review 51, no. 2: 214–227.

[ece370387-bib-0029] Hilton‐Taylor, C. 2000. “2000 IUCN Red List of Threatened Species.” Gland, Switzerland and Cambridge, UK: IUCN. https://portals.iucn.org/library/sites/library/files/documents/RL‐2000‐001.pdf.

[ece370387-bib-0030] HMGN (His Majesty's Government of Nepal) . 1996. Buffer Zone Management Regulation. Kathmandu, Nepal: Nepal Law Book Library.

[ece370387-bib-0031] Hoeks, S. , M. A. Huijbregts , M. Busana , M. B. Harfoot , J. C. Svenning , and L. Santini . 2020. “Mechanistic Insights Into the Role of Large Carnivores for Ecosystem Structure and Functioning.” Ecography 43, no. 12: 1752–1763.

[ece370387-bib-0205] Hotelling, H. 1936. “Relations Between Two Sets of Variates.” Biometrika 28, no. 3/4: 321.

[ece370387-bib-0033] Inskip, C. , and A. Zimmermann . 2009. “Human‐Felid Conflict: A Review of Patterns and Priorities Worldwide.” Oryx 43, no. 1: 18–34.

[ece370387-bib-0034] Kandel, S. R. , B. R. Lamichhane , and N. Subedi . 2020. “Leopard (*Panthera pardus*) Density and Diet in a Forest Corridor of Terai: Implications for Conservation and Conflict Management.” Wildlife Research 47, no. 6: 460–467.

[ece370387-bib-0035] Kapfer, P. M. , H. M. Streby , B. Gurung , A. Simcharoen , C. C. McDougal , and J. L. Smith . 2011. “Fine‐Scale Spatio‐Temporal Variation in Tiger Panthera Tigris Diet: Effect of Study Duration and Extent on Estimates of Tiger Diet in Chitwan National Park, Nepal.” Wildlife Biology 17, no. 3: 277–285.

[ece370387-bib-0036] Karra, K. , C. Kontgis , Z. Statman‐Weil , J. C. Mazzariello , M. Mathis , and S. P. Brumby . 2021. Global Land Use/Land Cover With Sentinel 2 and Deep Learning, 4704–4707. Brussels, Belgium: IEEE International Geoscience and Remote Sensing Symposium IGARSS.

[ece370387-bib-0037] Kelchtermans, S. 2020. Diet Study of the Tiger (Panthera tigris tigris) in Chitwan National Park, Nepal, With Specific Focus on the Buffer Zone and the Surrounding Areas, in Relation to Human‐Wildlife Conflicts: A Thesis Presented in Partial Fulfillment of the Requirements for the Degree of M.Sc. in Biodiversity. Belgium: Antwerp Univeristy.

[ece370387-bib-0038] Lamichhane, B. R. , H. Leirs , G. A. Persoon , et al. 2019. “Factors Associated With Co‐Occurrence of Large Carnivores in a Human‐Dominated Landscape.” Biodiversity and Conservation 28: 1473–1491.

[ece370387-bib-0039] Lamichhane, B. R. , G. A. Persoon , H. Leirs , et al. 2018. “Spatio‐Temporal Patterns of Attacks on Human and Economic Losses From Wildlife in Chitwan National Park, Nepal.” PLoS One 13, no. 4: e0195373. 29672538 10.1371/journal.pone.0195373 PMC5908188

[ece370387-bib-0040] Lamichhane, S. , and B. R. Jha . 2015. “Prey Selection by Bengal Tiger *Panthera tigris* tigris (Mammalia: Carnivora: Felidae) of Chitwan National Park, Nepal.” Journal of Threatened Taxa 7, no. 14: 8081–8088.

[ece370387-bib-0041] Lamichhane, S. , B. R. Lamichhane , A. Gurung , et al. 2023. “Non‐Exploitative Human Disturbance Provides Shelter for Prey From Predator.” Ecology and Evolution 13, no. 6: e10200. 37332517 10.1002/ece3.10200 PMC10269119

[ece370387-bib-0042] Leibold, M. A. , J. M. Chase , J. B. Shurin , and A. L. Downing . 1997. “Species Turnover and the Regulation of Trophic Structure.” Annual Review of Ecology and Systematics 28, no. 1: 467–494.

[ece370387-bib-0043] Lovari, S. , C. P. Pokheral , S. R. Jnawali , L. Fusani , and F. Ferretti . 2015. “Coexistence of the Tiger and the Common Leopard in a Prey‐Rich Area: The Role of Prey Partitioning.” Journal of Zoology 295, no. 2: 122–131.

[ece370387-bib-0044] Mardia, K. V. , J. T. Kent , and J. M. Bibby . 1979. Multivariate Analysis. London: Academic Press.

[ece370387-bib-0045] Marquet, P. A. , R. A. Quiñones , S. Abades , et al. 2005. “Scaling and Power‐Laws in Ecological Systems.” Journal of Experimental Biology 208, no. 9: 1749–1769. 15855405 10.1242/jeb.01588

[ece370387-bib-0046] McCauley, E. , and J. Kalff . 1981. “Empirical Relationships Between Phytoplankton and Zooplankton Biomass in Lakes.” Canadian Journal of Fisheries and Aquatic Sciences 38, no. 4: 458–463.

[ece370387-bib-0047] Mondal, K. , S. Gupta , S. Bhattacharjee , Q. Qureshi , and K. Sankar . 2012. “Response of Leopards to Re‐Introduced Tigers in Sariska Tiger Reserve, Western India.” International Journal of Biodiversity and Conservation 4, no. 5: 228–236.

[ece370387-bib-0048] Odden, M. , P. Wegge , and T. Fredriksen . 2010. “Do Tigers Displace Leopards? If So, Why?” Ecological Research 25: 875–881.

[ece370387-bib-0049] Oksanen, L. , and T. Oksanen . 2000. “The Logic and Realism of the Hypothesis of Exploitation Ecosystems.” American Naturalist 155, no. 6: 703–723. 10.1086/303354 10805639

[ece370387-bib-0050] Perkins, D. M. , I. A. Hatton , B. Gauzens , et al. 2022. “Consistent Predator–Prey Biomass Scaling in Complex Food Webs.” Nature Communications 13, no. 1: 4990. 10.1038/s41467-022-32578-5 PMC9411528 36008387

[ece370387-bib-0051] Plummer, M. 2015. “Package ‘rjags’.” Extracted on 13 December 2023. https://cran.r‐project.org/web/packages/rjags/rjags.pdf.

[ece370387-bib-0052] PNP . 2018. Parsa National Park and Its Buffer Zone Management Plan (FY2075/76‐2079/80). Kathmandu, Nepal: Department of National Parks and Wildlife Conservation. Ministry of Forests and Environment.

[ece370387-bib-0053] Pokheral, C. P. , and P. Wegge . 2019. “Coexisting Large Carnivores: Spatial Relationships of Tigers and Leopards and Their Prey in a Prey‐Rich Area in Lowland Nepal.” Ecoscience 26, no. 1: 1–9.

[ece370387-bib-0054] Pun, P. , S. Lamichhane , D. R. Thanet , P. R. Regmi , A. Maharjan , and B. R. Lamichhane . 2022. “Dietary Composition and Prey Preference of Royal Bengal Tiger (*Panthera tigris tigris*, Linnaeus 1758) of Parsa National Park, Nepal.” European Journal of Ecology 8, no. 1: 38–48.

[ece370387-bib-0056] Reddy, H. S. , C. Srinivasulu , and K. T. Rao . 2004. “Prey Selection by the Indian Tiger (*Panthera tigris tigris*) in Nagarjunasagar Srisailam Tiger Reserve, India.” Mammalian Biology 69, no. 6: 384–391.

[ece370387-bib-0057] Reimoser, F. , and R. Putman . 2011. Impacts of Wild Ungulates on Vegetation: Costs and Benefits, 144–191. Cambridge: Ungulate Management in Europe: Problems and Practices. Cambridge University Press.

[ece370387-bib-0058] Ripple, W. J. , J. A. Estes , R. L. Beschta , et al. 2014. “Status and Ecological Effects of the World's Largest Carnivores.” Science 343, no. 6167: 1241484. 24408439 10.1126/science.1241484

[ece370387-bib-0059] Seidensticker, J. 1976. “On the Ecological Separation Between Tigers and Leopards.” Biotropica 8: 225–234.

[ece370387-bib-0060] ShNP . 2023. Management Plan of Shuklaphanta National Park and Its Buffer Zone (FY2080/81‐2084/2085). Kathmandu, Nepal: Department of National Parks and Wildlife Conservation. Ministry of Forests and Environment.

[ece370387-bib-0061] Thapa, K. , R. Shrestha , J. Karki , et al. 2014. “Leopard *Panthera pardus fusca* Density in the Seasonally Dry, Subtropical Forest in the Bhabhar of Terai Arc, Nepal.” Advances in Ecology 2014, no. 1: 286949.

[ece370387-bib-0062] Trebilco, R. , J. K. Baum , A. K. Salomon , and N. K. Dulvy . 2013. “Ecosystem Ecology: Size‐Based Constraints on the Pyramids of Life.” Trends in Ecology & Evolution 28, no. 7: 423–431. 23623003 10.1016/j.tree.2013.03.008

[ece370387-bib-0063] Valente, A. M. , P. Acevedo , A. M. Figueiredo , C. Fonseca , and R. T. Torres . 2020. “Overabundant Wild Ungulate Populations in Europe: Management With Consideration of Socio‐Ecological Consequences.” Mammal Review 50, no. 4: 353–366.

[ece370387-bib-0064] Wegge, P. , M. Odden , C. P. Pokharel , and T. Storaas . 2009. “Predator–Prey Relationships and Responses of Ungulates and Their Predators to the Establishment of Protected Areas: A Case Study of Tigers, Leopards and Their Prey in Bardia National Park, Nepal.” Biological Conservation 142, no. 1: 189–202.

[ece370387-bib-0065] Wolf, C. , and W. J. Ripple . 2016. “Prey Depletion as a Threat to the World's Large Carnivores.” Royal Society Open Science 3, no. 8: 160252. 27853599 10.1098/rsos.160252 PMC5108949

[ece370387-bib-0066] Wolf, C. , and W. J. Ripple . 2018. “Rewilding the World's Large Carnivores.” Royal Society Open Science 5, no. 3: 172235. 29657815 10.1098/rsos.172235 PMC5882739

